# SLAM Project - Long Term Ecological Study of the Impacts of Climate Change in the natural forest of Azores: VI - Inventory of Arthropods of Azorean Urban Gardens

**DOI:** 10.3897/BDJ.11.e98286

**Published:** 2023-01-19

**Authors:** Lucas Lamelas-Lopez, Rosalina Gabriel, Alejandra Ros-Prieto, Paulo A. V. Borges

**Affiliations:** 1 cE3c- Centre for Ecology, Evolution and Environmental Changes, Azorean Biodiversity Group, CHANGE – Global Change and Sustainability Institute, Faculty of Agricultural Sciences and Environment, University of the Azores, Rua Capitão João d´Ávila, Pico da Urze, 9700-042, Angra do Heroísmo, Azores, Portugal cE3c- Centre for Ecology, Evolution and Environmental Changes, Azorean Biodiversity Group, CHANGE – Global Change and Sustainability Institute, Faculty of Agricultural Sciences and Environment, University of the Azores, Rua Capitão João d´Ávila, Pico da Urze, 9700-042 Angra do Heroísmo, Azores Portugal; 2 IUCN SSC Mid-Atlantic Island Invertebrate Specialist Group, Angra do Heroísmo, Azores, Portugal IUCN SSC Mid-Atlantic Island Invertebrate Specialist Group Angra do Heroísmo, Azores Portugal

**Keywords:** arthropods, biodiversity, dataset, inventory, introduced species, native species, Oceanic Islands, urban gardens

## Abstract

**Background:**

The data we present are part of the long-term project SLAM (Long Term Ecological Study of the Impacts of Climate Change in the natural forest of Azores) aiming to assess the impact of biodiversity erosion drivers on Azorean native biota, using long-term ecological data. Additionally to SLAM (Sea, Land and Air Malaise) traps, nocturnal Active Aerial Searching and nocturnal Foliage Beating methods were used to sample, between 2017 and 2018, the arthropod biodiversity on two historical urban gardens of Azores, the “Jardim Botânico” of Faial Island and “Jardim Duque da Terceira” of Terceira Island.

**New information:**

We provided an inventory of arthropods collected between 2017 and 2018 in two urban gardens of Faial and Terceira Islands (Azores). A total of 8342 specimens were collected, in which 7493 specimens were identified to species/subspecies level (Faial n = 3296; Terceira n = 4197). The identified specimens belong to four classes, 15 orders, 80 families and 159 species and subspecies of arthropods. A total of 84 species and subspecies are considered introduced (n = 2454 specimens), 50 native non-endemic (n = 4444 specimens), eight endemic (n = 217) and 17 have an indeterminate origin (n = 378). This study also revises the arthropod inventory of these Azorean gardens, by adding/updating the taxonomic names of three orders, ten families and 22 species.

## Introduction

Habitat loss, associated with landscape transformation, is one of the major causes of biodiversity loss worldwide (Diamond et al. 1989, Ntshanga et al. 2021). Particularly, the urbanisation process radically modifies the ecology of natural landscapes (Tratalos et al. 2007, Goddard et al. 2010). In addition to habitat loss, urbanisation also facilitates the introduction and establishment of exotic species and can affect the ecological interactions between local species (McKinney 2006).

In this context, urban gardens may play an important role in biodiversity conservation by provisioning a refuge for native biota and mitigating the effects of landscape fragmentation (Smith et al. 2005, Fuller et al. 2007, Goddard et al. 2010, Arteaga et al. 2020). Although the design and planning of urban gardens can affect positively native biodiversity, many urban gardens include exotic plant species that could facilitate the establishment of generalist introduced species (Matteson et al. 2008, Kowarik 2011).

This study complements the publication of Arteaga et al. (2020), which provides an inventory of arthropod diversity in Azorean urban gardens and studies the effect of plant species composition in the colonisation status of arthropods. Arteaga et al. (2020) demonstrated that, in general, arthropod communities are related with the plant species composition of gardens. More endemic and native arthropod species are found in gardens dominated by native plants, in comparison with gardens dominated by ornamental exotic plant species, where the proportion of introduced arthropods (individuals and species) was higher.

## General description

### Purpose

The main objective of this publication is to provide a recent inventory of the arthropod diversity present in two historical gardens of Azores, the “Jardim Botânico” of Faial Island and “Jardim Duque da Terceira” of Terceira Island, complementing the work of Arteaga et al. (2020). This study also updates the taxonomic inventory of Arteaga et al. (2020) and contributes to the study of the urban garden’s role in the conservation of native biodiversity.

### Additional information

The data we present are part of the long-term project SLAM (Long Term Ecological Study of the Impacts of Climate Change in the natural forest of Azores) aiming to assess the impact of biodiversity erosion drivers on Azorean native biota, using long-term ecological data.

This is the sixth dataset contribution for this project (previous ones in Costa and Borges (2021), Borges et al. (2022b), Borges et al. (2022a), Lhoumeau et al. (2022), Lhoumeau and Borges (2022)). Another publication dedicated to Lepidoptera contributed with information about some new exotic species for Azores (Pérez Santa-Rita et al. 2018). However, in the current study, additional sampling methods were also used, to include Active Aerial Searching and nocturnal Foliage Beating (see more details below).

## Project description

### Title

Inventory of Arthropods of Azorean Urban Gardens.

### Personnel

The project was conceived and is being led by Paulo A.V. Borges.

**Fieldwork**:

Terceira Island: Paulo A.V. Borges, Rosalina Gabriel, Alejandra Ros-Prieto.

Faial Island: Paulo A.V. Borges, Rosalina Gabriel, Pedro Casimiro.

**Parataxonomists**: Alejandra Ros-Prieto, Alba Arteaga.

**Taxonomists**: Paulo A. V. Borges and Luís Carlos Crespo.

**Curation**: Voucher specimen management was mainly undertaken by Alejandra Ros-Prieto, Alba Arteaga, Lucas Lamelas-López and Paulo A. V. Borges.

### Study area description

The study area comprises Terceira (total area: 400.2 km²; maximum elevation: 1021 m a.s.l.) and Faial (total area: 172 km2; maximum elevation 1043 m a.s.l.) Islands. They are located in the central group of the Azores Archipelago (North Atlantic), roughly at: 38°43′40″N, 27°12′48″W (Terceira Island), and 38°34′57″N, 28°42′17″W (Faial Island). The climate of the Archipelago is temperate oceanic, characterised by regular and abundant rainfall, high levels of relative humidity and persistent winds. The landscape of the Islands is mainly dominated by urban and agricultural areas at the lowest elevations; pasturelands and exotic tree plantations inland; and native forests located at highest elevations (Gaspar et al. 2010). The study was carried out on two botanical gardens, named “Jardim Botânico”, in Faial Island and “Jardim Duque da Terceira” in Terceira Island.

The Faial Island Botanical Garden (“Jardim Botânico”) was initially implemented in 1986 with the aim to promote the conservation of the flora of the Azores (Melo 2020). Initially occupying an area of 5,600 m², it is located in the parish of Flamengos, at an altitude of 118 m (Melo 2020). Additional terrain was added in the last decades and now it occupies 15,000 m² (1.5 ha) (Melo 2020). This is currently an iconic place in Faial Island visited by many tourists. In addition to a large collection of native and endemic plants, in 2003, this Boatnical Garden created the "Azores Seed Bank", whose purpose is to collect and maintain a collection of viable seeds of all Azorean species that are possible to conserve in a conventional seed bank (Melo 2020).

The “Jardim Duque da Terceira” in Terceira Island is located in the historic centre of the main town, Angra do Heroísmo, at an altitude of 34 m. Initially occupying an area of 16,000 m² in 1882, it now occupies a larger area that reaches 2 ha (Barcelos 2012). This Garden is dominated by exotic plants, transported to the Island since the period of the Portuguese discoveries and includes both tropical and subtropical species (Barcelos 2012).

### Design description

**Figure 1.**
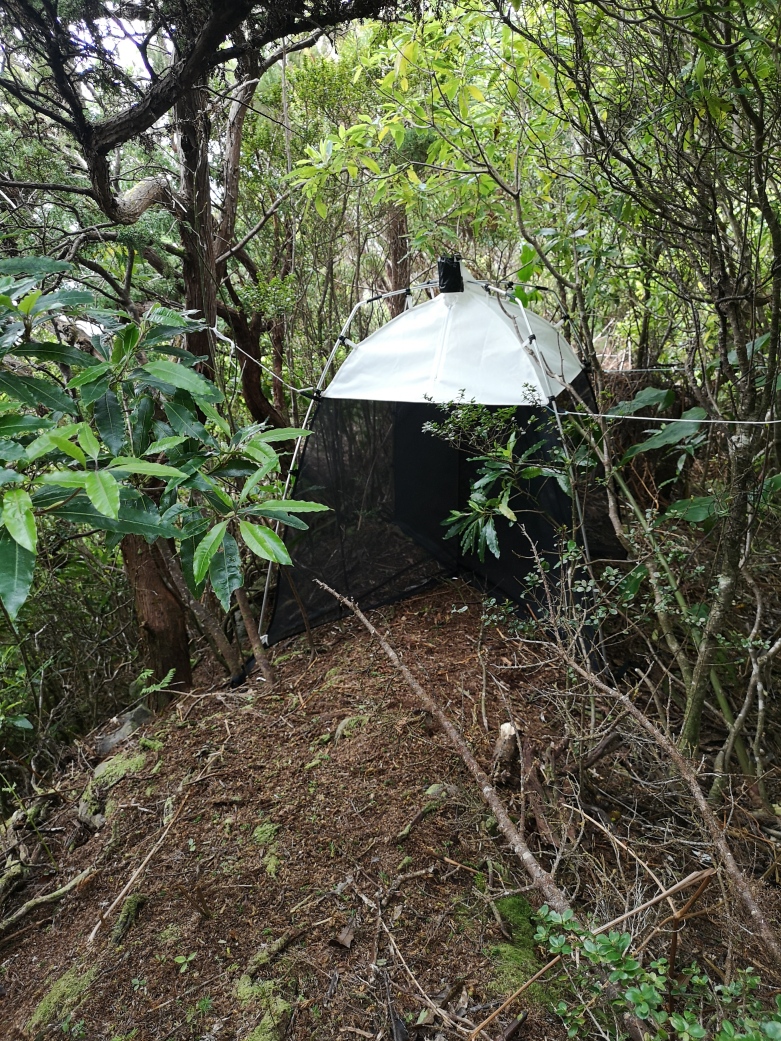
SLAM trap (Sea, Land and Air Malaise trap) located in a site on Terceira Island (Credit: Paulo A. V. Borges)

Passive Flight Interception traps (SLAM traps - Sea, Land and Air Malaise) (Fig. 1), nocturnal Active Aerial Searching (AAS) and nocturnal Foliage Beating (FBN) methods were used to sample the arthropod biodiversity on two historical urban gardens of Azores: the “Jardim Botânico”, located in the surroundings of Horta, in Faial Island and “Jardim Duque da Terceira” located in Angra do Heroísmo, in Terceira Island. AAS and FBN are reliable methods to collect samples of arthropods that are mainly active during the night (Borges et al. 2018). The collected specimens were preserved in ethanol 96%. SLAM traps were placed in both gardens in order to collect mainly diurnal flying and non-flying arthropods, through interception and conservation on a propylene-glycol recipient of the captured specimens (Borges et al. 2017). The SLAM traps were placed during six consecutive months and checked monthly.

### Funding

Fieldwork: FEDER in 85% and by Azorean Public funds by 15% through Operational Programme Azores 2020, under the project Green Garden Azores (ACORES-01-0145-FEDER-000070).

Taxomomic work: FEDER in 85% and by Azorean Public funds by 15% through Operational Programme Azores 2020, under the project AZORESBIOPORTAL (ACORES-01-0145-FEDER-000072) and also the project Portal da Biodiversidade dos Açores (2022-2023) - PO Azores Project - M1.1.A/INFRAEST CIENT/001/2022.

Data curation (Darwin Core): MACRISK-Trait-based prediction of extinction risk and invasiveness for Northern Macaronesian arthropods (FCT-PTDC/BIA-CBI/0625/2021).

## Sampling methods

### Study extent

The study was conducted on two urban gardens, the “Jardim Botânico”, located in the surroundings of Horta, in Faial Island and “Jardim Duque da Terceira” located in Angra do Heroísmo, in Terceira Island. The first is mainly composed of endemic and native plant species, but also includes some introduced species, common and widespread in the Azores. The second garden includes mainly collections of introduced trees, shrubs and palms from across the world (see for more details, Arteaga et al. (2020)).

### Sampling description

Passive Flight Interception traps (SLAM traps - Sea, Land and Air Malaise trap) (Fig. 1), nocturnal Active Aerial Searching (AAS) and nocturnal Foliage Beating (FBN) methods were used to sample the arthropod biodiversity (Arachnida, Chilopoda, Diplopoda and Insecta Classes) on two historical urban gardens of the Azores, between 2017 and 2018: the “Jardim Botânico”, located in Horta, in Faial Island and “Jardim Duque da Terceira”, located in Angra do Heroísmo, in Terceira Island. AAS consists on collecting arthropods found above knee-level by hand, forceps, pooter or brush and immediately transferring them into vials containing ethanol 96%. FBN consists of beating tree and shrub branches with a wooden stick and collecting the fallen specimens on a beating tray, posteriorly transferred to vials containing ethanol 96%. AAS and FBN are reliable methods to collect samples of arthropods that are mainly active during the night (Borges et al. 2018). The SLAM trap consists on a structure of 110 × 110 × 110 cm (MegaView Science Co.) designed to intercept flying and non-flying arthropods. They were placed in the gardens during six consecutive months, checked monthly. For more details about sampling methods, see Arteaga et al. (2020).

### Quality control

All collected specimens were sorted and posteriorly identified by an expert taxonomist (P.A.V.B) in the laboratory.

## Geographic coverage

### Description

Faial and Terceira Islands, Azores, Portugal

### Coordinates

38.508 and 38.807 Latitude; -28.839 and -27.0389 Longitude.

## Taxonomic coverage

### Description

The following Classes and Orders are covered:

Arachnida: Araneae; Opiliones; Pseudoscorpiones.

Chilopoda: Scutigeromorpha.

Diplopoda: Julida.

Insecta: Archaeognatha; Blattodea; Coleoptera; Dermaptera; Hemiptera; Hymenoptera; Neuroptera; Phasmida; Psocodea; Thysanoptera.

## Temporal coverage

### Notes

The data were collected between April 2017 and 30 June 2018.

## Collection data

### Collection name

Entomoteca Dalberto Teixeira Pombo at University of the Azores.

### Collection identifier

DTP

### Specimen preservation method

Alcohol

## Usage licence

### Usage licence

Creative Commons Public Domain Waiver (CC-Zero)

## Data resources

### Data package title

Inventory of Arthropods of Azorean Urban Gardens

### Resource link

http://ipt.gbif.pt/ipt/resource?r=arthropods_azorean_urban_gardens

### Alternative identifiers

https://www.gbif.org/dataset/3c314464-509f-4971-80d7-cd9f02110ea7

### Number of data sets

2

### Data set 1.

#### Data set name

Event Table

#### Data format

Darwin Core Archive format

#### Character set

UTF-8

#### Download URL

http://ipt.gbif.pt/ipt/resource?r=arthropods_azorean_urban_gardens

#### Data format version

1.5

#### Description

The dataset was published in the Global Biodiversity Information Facility platform, GBIF (Borges and Lamelas-López 2022). The following data table includes all the records for which a taxonomic identification of the species was possible. The dataset submitted to GBIF is structured as a sample event dataset that has been published as a Darwin Core Archive (DwCA), which is a standardised format for sharing biodiversity data as a set of one or more data tables. The core data file contains 20 records (eventID). This GBIF IPT (Integrated Publishing Toolkit, Version 2.5.6) archives the data and, thus, serves as the data repository. The data and resource metadata are available for download in the Portuguese GBIF Portal IPT (Borges and Lamelas-López 2022).

**Data set 1.** 

Column label	Column description
eventID	Identifier of the events, unique for the dataset.
stateProvince	Name of the region of the sampling site.
islandGroup	Name of the archipelago.
island	Name of the island.
country	Country of the sampling site.
countryCode	ISO code of the country of the sampling site.
municipality	Municipality of the sampling site.
locality	Locality of the sampling site.
locationID	Identifier of the location.
habitat	The habitat of the sampling site.
decimalLongitude	The geographic longitude (in decimal degrees, using the spatial reference system given in geodeticDatum) of the geographic centre of a Location.
decimalLatitude	The geographic latitude (in decimal degrees, using the spatial reference system given in geodeticDatum) of the geographic centre of a Location.
geodeticDatum	The ellipsoid, geodetic datum or spatial reference system (SRS) upon which the geographic coordinates given in decimalLatitude and decimalLongitude are based.
coordinateUncertaintyInMetres	Uncertainty of the coordinates of the centre of the sampling plot in metres.
coordinatePrecision	A decimal representation of the precision of the coordinates given in the decimalLatitude and decimalLongitude.
georeferenceSources	A list (concatenated and separated) of maps, gazetteers or other resources used to georeference the Location, described specifically enough to allow anyone in the future to use the same resources.
minimumElevationInMetres	The lower limit of the range of elevation (altitude, above sea level), in metres.
samplingProtocol	The sampling protocol used to capture the species.
sampleSizeValue	The numeric amount of time spent in each sampling.
sampleSizeUnit	The unit of the sample size value.
eventDate	Date or date range the record was collected.
year	Year of the event.
month	Month of the event.
day	Day of the event.

### Data set 2.

#### Data set name

Occurrence_Table

#### Data format

Darwin Core Archive format

#### Character set

UTF-8

#### Download URL

http://ipt.gbif.pt/ipt/resource?r=arthropods_azorean_urban_gardens

#### Data format version

1.5

#### Description

The dataset was published in the Global Biodiversity Information Facility platform, GBIF (Borges and Lamelas-López 2022), structured as an occurrence table that has been published as a Darwin Core Archive (DwCA), which is a standardised format for sharing biodiversity data as a set of one or more data tables. The core data file contains 762 records (occurrenceID). This GBIF IPT (Integrated Publishing Toolkit, Version 2.5.6) archives the data and, thus, serves as the data repository. The data and resource metadata are available for download in the Portuguese GBIF Portal IPT (Borges and Lamelas-López 2022).

**Data set 2.** 

Column label	Column description
eventID	Identifier of the events, unique for the dataset.
type	Type of the record, as defined by the Public Core standard.
licence	Reference to the licence under which the record is published.
institutionID	The identity of the institution publishing the data.
institutionCode	The code of the institution publishing the data.
collectionID	The identity of the collection publishing the data.
collectionCode	The code of the collection where the specimens are conserved.
datasetName	Name of the dataset
basisOfRecord	The nature of the data record.
occurrenceID	Identifier of the record, coded as a global unique identifier.
recordedBy	A list (concatenated and separated) of names of people, groups or organisations who performed the sampling in the field.
identifiedBy	A list (concatenated and separated) of names of people, groups or organisations who performed the sampling in the field.
dateIdentified	The date on which the subject was determined as representing the Taxon.
organismQuantity	A number or enumeration value for the quantity of organisms.
organismQuantityType	The type of quantification system used for the quantity of organisms.
sex	The sex and quantity of the individuals captured.
lifeStage	The life stage of the organisms captured.
identificationRemarks	Information about morphospecies identification (code in Dalberto Teixeira Pombo Collection).
scientificName	Complete scientific name including author and year.
kingdom	Kingdom name.
phylum	Phylum name.
class	Class name.
order	Order name.
family	Family name.
genus	Genus name.
specificEpithet	Specific epithet.
infraspecificEpithet	Infraspecific epithet.
scientificNameAuthorship	Name of the author of the lowest taxon rank included in the record.
taxonRank	Lowest taxonomic rank of the record.
establishmentMeans	The process of establishment of the species in the location, using a controlled vocabulary: 'native', 'introduced', 'endemic', 'indeterminate'.

## Additional information

We collected a total of 8342 individuals in both urban gardens, in which 7493 specimens were identified to species/subspecies level (Faial n = 3296; Terceira n = 4197). The identified specimens belong to four classes, 15 orders, 80 families and 159 species and subspecies of arthropods. A total of 84 species and subspecies are considered introduced (n = 2454 specimens), 50 native non-endemic (n = 4444 specimens), eight endemic (n = 217) and 17 have an indeterminate origin (n = 378) (Table 1).

In general, the most abundant species were the barklice *Trichopsocusclarus* (Banks, 1908) (Psocodea, Trichopsocidae) (n = 1169), which were captured in both urban gardens (Faial n = 502; Terceira n = 667), the fulgoroid planthopper *Cyphopterumadcendens* (Herrich-Schäffer, 1835) (Hemiptera, Flatidae), recorded only in Faial urban garden (n = 725) and the ant *Lasiusgrandis* Forel, 1909 (Hymenoptera, Formicidae) (n = 555) being recorded in both Islands (Faial n = 101; Terceira n = 454; Table 2). These three species are considered native non-endemic in the Archipelago. The most common endemic species were the lacewing *Hemerobiusazoricus* Tjeder, 1948 (Neuroptera, Hemerobiidae) (n = 92) and the spider *Emblynaacoreensis* Wunderlich, 1992 (Araneae, Dictynidae) (n = 57), being more abundant in the Faial urban garden (n = 87 and n = 50, respectively), than in the Terceira urban garden (n = 5 and n = 7, respectively). The most abundant introduced species were the spider *Neosconacrucifera* (Lucas, 1838) (Araneae, Araneidae) (n = 331) and the true bug *Oxycarenuslavaterae* (Fabricius, 1787) (Hemiptera, Oxycarenidae) (n = 281), the first species being more abundant in Faial (n = 287) than in Terceira (n = 44) and the second one absent in Faial urban garden (Table 1). The most common recorded arthropod families were Flatidae (Hemiptera; n = 888) and Trichopsocidae (Psocodea; n = 1169), being relatively abundant in both urban gardens (Table 2).

Considering the identified taxa (Table 1), we recorded 72 species and subspecies in Faial, with 28 being considered native non-endemic, seven endemic, 33 introduced and four of indeterminate origin. On the other hand, in Terceira, a total of 124 species and subspecies were recorded, 37 being considered native non-endemic, five endemic, 67 introduced and 15 of indeterminate origin (Table 1). The proportion of native endemic and non-endemic species in Terceira urban garden (33.87%) is lower than in Faial (48.61%) and the proportion of introduced species is higher in Terceira urban garden (54.03%) in comparison with Faial (45.83%).

This study also updates the taxonomy of the arthropods of the Azorean urban gardens. A total of three orders, ten families and 22 species were taxonomically updated (Table 3).

This publication includes a recent inventory and updates the knowledge about the arthropod diversity and taxonomy of Arteaga et al. (2020). In general, the Terceira garden is mainly dominated by exotic plant species and, consequently, the proportion of introduced arthropods species is higher than in Faial, which is mainly composed by native plant species. Contrarily, the proportion of native species (endemic and non-endemic) is higher in Faial than in Terceira. These results are according to the findings of Arteaga et al. (2020).

Public and botanical gardens are important green infrastructures that promote the conservation of plants species, support science dissemination activities and people's health. Additional positive functions may include microclimatic regulation and water retention (Macháč et al. 2022). However, there is an ongoing debate on the role of gardens dominated by exotic plants and their role as a source for the spread of exotic potentially invasive species (Dawson et al. 2008). Concerning arthropods, our study generated several interesting patterns:

i) no introduced species had a dominant role in any garden, despite several being part of the 50% most abundant species in Terceira;

iii) in Faial Botanical Garden, the 50% most abundant species are either endemic or native non-endemic, with only one introduced species;

iii) most introduced and species of indeterminate status are particularly rare.

In conclusion, in general, the origin of the plant composition of the urban gardens can have an effect on the arthropod biodiversity origin (native vs. introduced species) present in the gardens, but the two studied settings also constitute a repository of indigenous fauna playing an important role in the conservation of native biota of the Archipelago. In particular, the Faial Island Botanical Garden, which holds a large community of native species, can be part of a future corridor of native plants across the agricultural landscape in this Island.

## Figures and Tables

**Table 1.** Inventory of arthropods recorded in Azorean urban gardens of “Jardim Botânico” of Faial Island (FAI) and “Jardim Duque da Terceira” of Terceira Island (TER), between 2017 and 2018. The colonisation status (C.S.: End – Endemic; Nat – Native non-endemic; Int – Introduced; Ind – Indeterminate) and abundance values per island and total are provided.

**Class**	**Order**	**Family**	**Scientific name**	**C.S.**	**FAI**	**TER**	**Total**
Arachnida	Araneae	Agelenidae	*Textrixcaudata* L. Koch, 1872	Int	10	0	10
Arachnida	Araneae	Araneidae	*Agalenatearedii* (Scopoli, 1763)	Int	0	11	11
Arachnida	Araneae	Araneidae	*Argiopebruennichi* (Scopoli, 1772)	Nat	0	2	2
Arachnida	Araneae	Araneidae	*Mangoraacalypha* (Walckenaer, 1802)	Int	1	0	1
Arachnida	Araneae	Araneidae	*Neosconacrucifera* (Lucas, 1838)	Int	287	44	331
Arachnida	Araneae	Araneidae	*Zygiellax-notata* (Clerck, 1757)	Int	8	2	10
Arachnida	Araneae	Cheiracanthiidae	*Cheiracanthiummildei* L. Koch, 1864	Int	2	0	2
Arachnida	Araneae	Clubionidae	*Clubionaterrestris* Westring, 1851	Int	2	0	2
Arachnida	Araneae	Clubionidae	*Porrhoclubionadecora* (Blackwall, 1859)	Nat	172	292	464
Arachnida	Araneae	Clubionidae	*Porrhoclubionagenevensis* (L. Koch, 1866)	Int	3	2	5
Arachnida	Araneae	Dictynidae	*Emblynaacoreensis* Wunderlich, 1992	End	50	7	57
Arachnida	Araneae	Dictynidae	*Nigmapuella* (Simon, 1870)	Int	13	15	28
Arachnida	Araneae	Linyphiidae	*Agynetafuscipalpa* (C. L. Koch, 1836)	Int	0	8	8
Arachnida	Araneae	Linyphiidae	*Entelecaraschmitzi* Kulczynski, 1905	Nat	71	4	75
Arachnida	Araneae	Linyphiidae	*Erigoneatra* Blackwall, 1833	Int	1	1	2
Arachnida	Araneae	Linyphiidae	*Erigoneautumnalis* Emerton, 1882	Int	0	1	1
Arachnida	Araneae	Linyphiidae	*Mermessusbryantae* (Ivie & Barrows, 1935)	Int	1	0	1
Arachnida	Araneae	Linyphiidae	*Mermessusfradeorum* (Berland, 1932)	Int	2	0	2
Arachnida	Araneae	Linyphiidae	*Microlinyphiajohnsoni* (Blackwall, 1859)	Nat	0	1	1
Arachnida	Araneae	Linyphiidae	*Nerieneclathrata* (Sundevall, 1830)	Int	1	1	2
Arachnida	Araneae	Linyphiidae	*Pelecopsisparallela* (Wider, 1834)	Int	1	1	2
Arachnida	Araneae	Linyphiidae	*Tenuiphantestenuis* (Blackwall, 1852)	Int	23	14	37
Arachnida	Araneae	Mimetidae	*Eroaphana* (Walckenaer, 1802)	Int	0	5	5
Arachnida	Araneae	Oecobiidae	*Oecobiusnavus* Blackwall, 1859	Int	0	1	1
Arachnida	Araneae	Pholcidae	*Pholcusphalangioides* (Fuesslin, 1775)	Int	0	2	2
Arachnida	Araneae	Salticidae	*Chalcoscirtusinfimus* (Simon, 1868)	Int	0	2	2
Arachnida	Araneae	Salticidae	*Macaroerisdiligens* (Blackwall, 1867)	Nat	0	17	17
Arachnida	Araneae	Salticidae	*Pseudeuophrysvafra* (Blackwall, 1867)	Int	0	10	10
Arachnida	Araneae	Salticidae	*Salticusmutabilis* Lucas, 1846	Int	0	3	3
Arachnida	Araneae	Tetragnathidae	*Metellinamerianae* (Scopoli, 1763)	Int	2	1	3
Arachnida	Araneae	Theridiidae	*Cryptachaeablattea* (Urquhart, 1886)	Int	15	4	19
Arachnida	Araneae	Theridiidae	*Dipoenaumbratilis* (Simon, 1873)	Int	23	0	23
Arachnida	Araneae	Theridiidae	*Paidiscuraorotavensis* (Schmidt, 1968)	Nat	0	15	15
Arachnida	Araneae	Theridiidae	*Parasteatodatepidariorum* (C. L. Koch, 1841)	Int	0	4	4
Arachnida	Araneae	Theridiidae	*Steatodagrossa* (C. L. Koch, 1838)	Int	43	0	43
Arachnida	Araneae	Theridiidae	*Steatodanobilis* (Thorell, 1875)	Nat	8	10	18
Arachnida	Araneae	Theridiidae	*Theridionhannoniae* Denis, 1945	Int	0	1	1
Arachnida	Araneae	Theridiidae	*Theridionmusivivum* Schmidt, 1956	Nat	2	0	2
Arachnida	Opiliones	Leiobunidae	*Leiobunumblackwalli* Meade, 1861	Nat	142	0	142
Arachnida	Pseudoscorpiones	Chthoniidae	*Chthoniusischnocheles* (Hermann, 1804)	Int	2	0	2
Arachnida	Pseudoscorpiones	Chthoniidae	*Ephippiochthoniustetrachelatus* (Preyssler, 1790)	Int	0	2	2
Chilopoda	Scutigeromorpha	Scutigeridae	*Scutigeracoleoptrata* (Linnaeus, 1758)	Int	0	71	71
Diplopoda	Julida	Julidae	*Ommatoiulusmoreleti* (Lucas, 1860)	Int	29	44	73
Insecta	Archaeognatha	Machilidae	*Diltasaxicola* (Womersley, 1930)	Nat	0	3	3
Insecta	Blattodea	Kalotermitidae	*Cryptotermesbrevis* (Walker, 1853)	Int	0	1	1
Insecta	Coleoptera	Apionidae	*Aspidapionradiolus* (Marsham, 1802)	Int	6	8	14
Insecta	Coleoptera	Apionidae	*Kalcapionsemivittatumsemivittatum* (Gyllenhal, 1833)	Ind	4	85	89
Insecta	Coleoptera	Carabidae	*Dromiusmeridionalis* Dejean, 1825	Int	3	0	3
Insecta	Coleoptera	Chrysomelidae	*Chaetocnemahortensis* (Fourcroy, 1785)	Int	0	62	62
Insecta	Coleoptera	Chrysomelidae	*Epitrixcucumeris* (Harris, 1851)	Int	0	172	172
Insecta	Coleoptera	Chrysomelidae	*Epitrixhirtipennis* (Melsheimer, 1847)	Int	0	4	4
Insecta	Coleoptera	Chrysomelidae	*Longitarsuskutscherai* (Rye, 1872)	Int	25	0	25
Insecta	Coleoptera	Chrysomelidae	*Psylliodesmarcida* (Illiger, 1807)	Nat	0	2	2
Insecta	Coleoptera	Coccinellidae	*Clitostethusarcuatus* (Rossi, 1794)	Int	0	7	7
Insecta	Coleoptera	Coccinellidae	*Scymniscushelgae* (Fürsch, 1965)	Int	0	13	13
Insecta	Coleoptera	Coccinellidae	*Scymnusinterruptus* (Goeze, 1777)	Nat	0	162	162
Insecta	Coleoptera	Coccinellidae	*Stethoruspusillus* (Herbst, 1797)	Nat	0	20	20
Insecta	Coleoptera	Corylophidae	*Sericoderuslateralis* (Gyllenhal, 1827)	Int	9	263	272
Insecta	Coleoptera	Cryptophagidae	*Cryptophaguscellaris* (Scopoli, 1763)	Int	0	2	2
Insecta	Coleoptera	Curculionidae	*Calacallessubcarinatus* (Israelson, 1984)	End	1	0	1
Insecta	Coleoptera	Curculionidae	*Coccotrypescarpophagus* (Hornung, 1842)	Int	0	69	69
Insecta	Coleoptera	Curculionidae	*Derelomuspiriformis* (Hoffmann, 1938)	Int	0	1	1
Insecta	Coleoptera	Curculionidae	*Lixuspulverulentus* (Scopoli, 1763)	Int	0	4	4
Insecta	Coleoptera	Curculionidae	*Mecinuspascuorum* (Gyllenhal, 1813)	Int	0	125	125
Insecta	Coleoptera	Curculionidae	*Naupactuscervinus* (Boheman, 1840)	Int	0	3	3
Insecta	Coleoptera	Curculionidae	*Naupactusleucoloma* Boheman, 1840	Int	0	11	11
Insecta	Coleoptera	Curculionidae	*Otiorhynchuscribricollis* Gyllenhal, 1834	Int	1	0	1
Insecta	Coleoptera	Curculionidae	*Sirocalodesmixtus* (Mulsant & Rey, 1859)	Int	0	3	3
Insecta	Coleoptera	Curculionidae	*Sitonacinnamomeus* Allard, 1863	Int	0	1	1
Insecta	Coleoptera	Dryophthoridae	*Sitophilusoryzae* (Linnaeus, 1763)	Int	0	1	1
Insecta	Coleoptera	Elateridae	*Heteroderesazoricus* (Tarnier, 1860)	End	2	1	3
Insecta	Coleoptera	Elateridae	*Heteroderesvagus* Candèze, 1893	Int	0	1	1
Insecta	Coleoptera	Latridiidae	*Cartoderebifasciata* (Reitter, 1877)	Int	1	28	29
Insecta	Coleoptera	Latridiidae	*Cartoderenodifer* (Westwood, 1839)	Int	0	4	4
Insecta	Coleoptera	Mycetophagidae	*Litargusbalteatus* LeConte, 1856	Int	0	12	12
Insecta	Coleoptera	Mycetophagidae	*Typhaeastercorea* (Linnaeus, 1758)	Int	0	7	7
Insecta	Coleoptera	Nitidulidae	*Phenolialimbatatibialis* (Boheman, 1851)	Int	0	2	2
Insecta	Coleoptera	Phalacridae	*Stilbustestaceus* (Panzer, 1797)	Nat	0	68	68
Insecta	Coleoptera	Ptiliidae	*Ptenidiumpusillum* (Gyllenhal, 1808)	Int	0	2	2
Insecta	Coleoptera	Ptinidae	*Anobiumpunctatum* (De Geer, 1774)	Int	0	6	6
Insecta	Coleoptera	Scraptiidae	*Anaspisproteus* Wollaston, 1854	Nat	1	0	1
Insecta	Coleoptera	Silvanidae	*Cryptamorphadesjardinsii* (Guérin-Méneville, 1844)	Int	0	2	2
Insecta	Coleoptera	Staphylinidae	*Athetafungi* (Gravenhorst, 1806)	Ind	0	62	62
Insecta	Coleoptera	Staphylinidae	*Carpelimuscorticinus* (Gravenhorst, 1806)	Ind	0	5	5
Insecta	Coleoptera	Staphylinidae	*Carpelimuszealandicus* (Sharp, 1900)	Int	0	1	1
Insecta	Coleoptera	Staphylinidae	*Coproporuspulchellus* (Erichson, 1839)	Ind	0	6	6
Insecta	Coleoptera	Staphylinidae	*Cordaliaobscura* (Gravenhorst, 1802)	Ind	0	3	3
Insecta	Coleoptera	Staphylinidae	*Hypomedondebilicornis* (Wollaston, 1857)	Ind	0	11	11
Insecta	Coleoptera	Staphylinidae	*Myrmecocephalusconcinnus* (Erichson, 1839)	Ind	0	1	1
Insecta	Coleoptera	Staphylinidae	*Oligotapumilio* Kiesenwetter, 1858	Ind	0	14	14
Insecta	Coleoptera	Staphylinidae	*Oxypodalurida* Wollaston, 1857	Ind	0	1	1
Insecta	Coleoptera	Staphylinidae	*Proteinusatomarius* Erichson, 1840	Ind	0	53	53
Insecta	Coleoptera	Staphylinidae	*Rugilusorbiculatus* (Paykull, 1789)	Ind	0	3	3
Insecta	Coleoptera	Staphylinidae	*Scopaeusportai* Luze, 1910	Ind	0	1	1
Insecta	Coleoptera	Staphylinidae	*Stenomastaxmadeirae* Assing, 2003	Ind	0	1	1
Insecta	Coleoptera	Staphylinidae	*Suniuspropinquus* (Brisout de Barneville, 1867)	Ind	1	0	1
Insecta	Coleoptera	Staphylinidae	*Tachyporuschrysomelinus* (Linnaeus, 1758)	Ind	18	37	55
Insecta	Coleoptera	Staphylinidae	*Tachyporusnitidulus* (Fabricius, 1781)	Ind	48	24	72
Insecta	Dermaptera	Anisolabididae	*Euborelliaannulipes* (Lucas, 1847)	Int	4	0	4
Insecta	Dermaptera	Forficulidae	*Forficulaauricularia* Linnaeus, 1758	Int	2	0	2
Insecta	Dermaptera	Labiduridae	*Labidurariparia* (Pallas, 1773)	Nat	4	0	4
Insecta	Dermaptera	Spongiphoridae	*Labiaminor* (Linnaeus, 1758)	Int	0	2	2
Insecta	Hemiptera	Anthocoridae	*Anthocorisnemoralis* (Fabricius, 1794)	Nat	0	11	11
Insecta	Hemiptera	Anthocoridae	*Buchananiellacontinua* (White, 1880)	Int	0	4	4
Insecta	Hemiptera	Anthocoridae	*Oriuslaevigatuslaevigatus* (Fieber, 1860)	Nat	2	14	16
Insecta	Hemiptera	Aphididae	*Cinarajuniperi* (De Geer, 1773)	Nat	374	0	374
Insecta	Hemiptera	Cicadellidae	*Eupteryxfilicum* (Newman, 1853)	Nat	5	15	20
Insecta	Hemiptera	Cicadellidae	*Euscelidiusvariegatus* (Kirschbaum, 1858)	Nat	0	40	40
Insecta	Hemiptera	Cicadellidae	*Sophoniaorientalis* (Matsumura, 1912)	Int	0	10	10
Insecta	Hemiptera	Cixiidae	*Cixiusazopifajoazofa* Remane & Asche, 1979	End	1	0	1
Insecta	Hemiptera	Delphacidae	*Kelisiaribauti* Wagner, 1938	Nat	0	5	5
Insecta	Hemiptera	Flatidae	*Cyphopterumadcendens* (Herrich-Schäffer, 1835)	Nat	725	0	725
Insecta	Hemiptera	Flatidae	*Siphantaacuta* (Walker, 1851)	Int	0	163	163
Insecta	Hemiptera	Liviidae	*Strophingiaharteni* Hodkinson, 1981	End	39	0	39
Insecta	Hemiptera	Lyctocoridae	*Lyctocoriscampestris* (Fabricius, 1794)	Int	0	2	2
Insecta	Hemiptera	Lygaeidae	*Kleidocerysericae* (Horváth, 1909)	Nat	20	2	22
Insecta	Hemiptera	Microphysidae	*Loriculacoleoptrata* (Fallén, 1807)	Nat	57	0	57
Insecta	Hemiptera	Miridae	*Campyloneuravirgula* (Herrich-Schaeffer, 1835)	Nat	37	0	37
Insecta	Hemiptera	Miridae	*Heterotomaplanicornis* (Pallas, 1772)	Nat	1	0	1
Insecta	Hemiptera	Miridae	*Monalocorisfilicis* (Linnaeus, 1758)	Nat	0	6	6
Insecta	Hemiptera	Miridae	*Pilophorusconfusus* (Kirschbaum, 1856)	Nat	37	19	56
Insecta	Hemiptera	Miridae	*Taylorilygusapicalis* (Fieber, 1861)	Int	0	2	2
Insecta	Hemiptera	Miridae	*Trigonotyluscaelestialium* (Kirkaldy, 1902)	Nat	0	7	7
Insecta	Hemiptera	Nabidae	*Nabispseudoferusibericus* Remane, 1962	Nat	0	1	1
Insecta	Hemiptera	Oxycarenidae	*Oxycarenuslavaterae* (Fabricius, 1787)	Int	0	281	281
Insecta	Hemiptera	Pentatomidae	*Nezaraviridula* (Linnaeus, 1758)	Int	0	1	1
Insecta	Hemiptera	Reduviidae	*Empicorisrubromaculatus* (Blackburn, 1889)	Int	14	7	21
Insecta	Hemiptera	Rhyparochromidae	*Aphanusrolandri* (Linnaeus, 1758)	Nat	0	4	4
Insecta	Hemiptera	Rhyparochromidae	*Beosusmaritimus* (Scopoli, 1763)	Nat	0	1	1
Insecta	Hemiptera	Rhyparochromidae	*Emblethisdenticollis* Horváth, 1878	Nat	0	1	1
Insecta	Hemiptera	Rhyparochromidae	*Scolopostethusdecoratus* (Hahn, 1833)	Nat	0	6	6
Insecta	Hemiptera	Triozidae	*Triozalaurisilvae* Hodkinson, 1990	Nat	21	0	21
Insecta	Hymenoptera	Formicidae	*Hypoponeraeduardi* (Forel, 1894)	Nat	4	0	4
Insecta	Hymenoptera	Formicidae	*Lasiusgrandis* Forel, 1909	Nat	101	454	555
Insecta	Hymenoptera	Formicidae	*Linepithemahumile* (Mayr, 1868)	Int	0	30	30
Insecta	Hymenoptera	Formicidae	*Monomoriumcarbonarium* (Smith, 1858)	Nat	0	5	5
Insecta	Hymenoptera	Formicidae	*Tetramoriumcaespitum* (Linnaeus, 1758)	Nat	0	18	18
Insecta	Hymenoptera	Formicidae	*Tetramoriumcaldarium* (Roger, 1857)	Int	0	14	14
Insecta	Neuroptera	Hemerobiidae	*Hemerobiusazoricus* Tjeder, 1948	End	87	5	92
Insecta	Phasmida	Phasmatidae	*Carausiusmorosus* (Sinéty, 1901)	Int	4	0	4
Insecta	Psocodea	Caeciliusidae	*Valenzuelaburmeisteri* (Brauer, 1876)	Nat	5	1	6
Insecta	Psocodea	Caeciliusidae	*Valenzuelaflavidus* (Stephens, 1836)	Nat	8	6	14
Insecta	Psocodea	Ectopsocidae	*Ectopsocusbriggsi* McLachlan, 1899	Int	16	50	66
Insecta	Psocodea	Ectopsocidae	*Ectopsocusstrauchi* Enderlein, 1906	Nat	1	90	91
Insecta	Psocodea	Elipsocidae	*Elipsocusazoricus* Meinander, 1975	End	18	5	23
Insecta	Psocodea	Elipsocidae	*Elipsocusbrincki* Badonnel, 1963	End	0	1	1
Insecta	Psocodea	Epipsocidae	*Bertkauialucifuga* (Rambur, 1842)	Nat	21	1	22
Insecta	Psocodea	Peripsocidae	*Peripsocusphaeopterus* (Stephens, 1836)	Nat	0	4	4
Insecta	Psocodea	Psocidae	*Atlantopsocusadustus* (Hagen, 1865)	Nat	98	5	103
Insecta	Psocodea	Trichopsocidae	*Trichopsocusclarus* (Banks, 1908)	Nat	502	667	1169
Insecta	Thysanoptera	Aeolothripidae	*Aeolothripsgloriosus* Bagnall, 1914	Nat	1	1	2
Insecta	Thysanoptera	Phlaeothripidae	*Hoplothripscorticis* (De Geer, 1773)	Nat	2	0	2
Insecta	Thysanoptera	Thripidae	*Ceratothripsericae* (Haliday, 1836)	Nat	42	0	42
Insecta	Thysanoptera	Thripidae	*Heliothripshaemorrhoidalis* (Bouché, 1833)	Int	8	3	11
Insecta	Thysanoptera	Thripidae	*Hercinothripsbicinctus* (Bagnall, 1919)	Int	1	245	246
Insecta	Thysanoptera	Thripidae	*Parthenothripsdracaenae* (Heeger, 1854)	Int	0	12	12

**Table 2.** Ranking of the ten most abundant species per urban garden. The colonisation statuses (C.S.: End – Endemic; Nat – Native non-endemic; Int – Introduced) and abundance values (N) are provided.

**Class**	**Order**	**Family**	**Scientific name**	**C.S.**	**N**
**Faial Urban Garden**					
Insecta	Hemiptera	Flatidae	*Cyphopterumadcendens* (Herrich-Schäffer, 1835)	Nat	725
Insecta	Psocodea	Trichopsocidae	*Trichopsocusclarus* (Banks, 1908)	Nat	502
Insecta	Hemiptera	Aphididae	*Cinarajuniperi* (De Geer, 1773)	Nat	374
Arachnida	Araneae	Araneidae	*Neosconacrucifera* (Lucas, 1838)	Int	287
Arachnida	Araneae	Clubionidae	*Porrhoclubionadecora* (Blackwall, 1859)	Nat	172
Arachnida	Opiliones	Leiobunidae	*Leiobunumblackwalli* Meade, 1861	Nat	142
Insecta	Hymenoptera	Formicidae	*Lasiusgrandis* Forel, 1909	Nat	101
Insecta	Psocodea	Psocidae	*Atlantopsocusadustus* (Hagen, 1865)	Nat	98
Insecta	Neuroptera	Hemerobiidae	*Hemerobiusazoricus* Tjeder, 1948	End	87
Arachnida	Araneae	Linyphiidae	*Entelecaraschmitzi* Kulczynski, 1905	Nat	71
**Terceira Urban Garden**					
Insecta	Psocodea	Trichopsocidae	*Trichopsocusclarus* (Banks, 1908)	Nat	667
Insecta	Hymenoptera	Formicidae	*Lasiusgrandis* Forel, 1909	Nat	454
Arachnida	Araneae	Clubionidae	*Porrhoclubionadecora* (Blackwall, 1859)	Nat	292
Insecta	Hemiptera	Oxycarenidae	*Oxycarenuslavaterae* (Fabricius, 1787)	Int	281
Insecta	Coleoptera	Corylophidae	*Sericoderuslateralis* (Gyllenhal, 1827)	Int	263
Insecta	Thysanoptera	Thripidae	*Hercinothripsbicinctus* (Bagnall, 1919)	Int	245
Insecta	Coleoptera	Chrysomelidae	*Epitrixcucumeris* (Harris, 1851)	Int	172
Insecta	Hemiptera	Flatidae	*Siphantaacuta* (Walker, 1851)	Int	163
Insecta	Coleoptera	Coccinellidae	*Scymnusinterruptus* (Goeze, 1777)	Nat	162
Insecta	Coleoptera	Curculionidae	*Mecinuspascuorum* (Gyllenhal, 1813)	Int	125

**Table 3.** Update of the taxonomy of the species recorded in the Azorean urban gardens of Faial and Terceira Islands. *Some species of Anthocoridae family change to Lyctocoridae; **Some species of Lygaeidae family change to Oxycarenidae and Rhyparochromidae; MF Morphospecies; *** - Not recorded in Arteaga et al. 2020.

**Level**	**Artega et al. (2020)**	**New Taxonomy**
Order	Psocoptera	Psocodea
Order	Microcoryphia	Archaeognatha
Order	Phasmatodea	Phasmida
Family	Eutichuridae	Cheiracanthiidae
Family	Phalangiidae	Leiobunidae
Family	Anobiidae	Ptinidae
Family	Brentidae	Apionidae
Family	Lathridiidae	Latridiidae
Family	Lachnidae	Aphididae
Family	Anthocoridae*	Lyctocoridae
Family	Lygaeidae**	Oxycarenidae
Family	Lygaeidae**	Rhyparochromidae
Family	Psyllidae	Liviidae
Species	*Meionetafuscipalpa* (C. L. Koch, 1836)	*Agynetafuscipalpa* (C. L. Koch, 1836)
Species	Carpelimus sp.	*Carpelimuszealandicus* (Sharp, 1900)
Species	MF 1376	*Derelomuspiriformis* (Hoffmann, 1938)
Species	Genus (?), species (?) ***	*Dipoenaumbratilis* (Simon, 1873)
Species	*Chthoniustetrachelatus* (Preyssler, 1790)	*Ephippiochthoniustetrachelatus* (Preyssler, 1790)
Species	*Kleidocerysericae* (Horváth, 1908)	*Kleidocerysericae* (Horváth, 1909)
Species	*Loriculaelegantula* (Bärensprung, 1858)	*Loriculacoleoptrata* (Fallén, 1807)
Species	*Gymnetronpascuorum* (Gyllenhal, 1813)	*Mecinuspascuorum* (Gyllenhal, 1813)
Species	*Monomoriumcarbonarium* (F. Smith, 1858)	*Monomoriumcarbonarium* (Smith, 1858)
Species	*Myrmecocephalusconcinnus* (Erichson, 1840)	*Myrmecocephalusconcinnus* (Erichson, 1839)
Species	*Pantomoruscervinus* (Boheman, 1849)	*Naupactuscervinus* (Boheman, 1840)
Species	MF 1385	*Oxypodalurida* Wollaston, 1857
Species	*Psylliodesmarcidus* (Illiger, 1807)	*Psylliodesmarcida* (Illiger, 1807)
Species	MF 551	*Scopaeusportai* Luze, 1910
Species	*Nephushelgae* Fürsch, 1965	*Scymniscushelgae* (Fürsch, 1965)
Species	*Sirocalodesmixtus* (Mulsant & Rey, 1858)	*Sirocalodesmixtus* (Mulsant & Rey, 1859)
Species	MF 1398	*Sitonacinnamomeus* Allard, 1863
Species	MF 1274	*Sophoniaorientalis* (Matsumura, 1912)
Species	*Stethoruspusillus* (Herbst, 1979)	*Stethoruspusillus* (Herbst, 1797)
Species	MF Formicidae F6	*Tetramoriumcaespitum* (Linnaeus, 1758)
Species	MF Formicidae F6	*Tetramoriumcaldarium* (Roger, 1857)
Species	*Theridionhannoniae* Denis, 1944	*Theridionhannoniae* Denis, 1945
